# Wedge Resection for Duodenal Gastrointestinal Stromal Tumors: Surgical Management and the Clinicopathological Outcome

**DOI:** 10.31662/jmaj.2021-0093

**Published:** 2021-12-03

**Authors:** Tetsunobu Udaka, Takeyoshi Nishiyama, Nobuyuki Watanabe, Izuru Endou, Osamu Yoshida, Hiroaki Asano, Masatoshi Kubo

**Affiliations:** 1 Department of Surgery, Mitoyo General Hospital, Kanonji, Japan

**Keywords:** Duodenum, GIST, Wedge resection, Prognosis, Surgery

## Abstract

We analyzed the clinicopathological characteristics of six patients with duodenal gastrointestinal stromal tumor (dGIST) resected in our hospital between 2005 and 2020. The patients (5 males, 1 female) were aged from 43 to 83 years old (mean: 63.7 years old). With respect to the preoperative diagnosis, one patient was diagnosed with dGIST by a biopsy, and five patients were diagnosed with suspected dGIST by esophagogastroduodenoscopy (EGD). The tumor locations were the third portion in four cases, second portion in one, and fourth portion in one. The pathological stages were I in four patients, II in one, and IIIB in one. All patients were discharged 12.8 days (10-15 days) postoperatively without complications, such as pancreatic fistula or suture deficiency. Regarding the prognosis, all patients are alive without recurrence.

The wedge resection is a reasonable option for resection of dGIST and should be routinely considered if technically feasible.

## Introduction

Duodenal gastrointestinal stromal tumors (dGISTs) are a very rare presentation, accounting for 4%-5% of all GIST, but are 6%-21% of surgically resected ones ^(1)^.

Clinically adopted surgical excision modalities for dGIST include pancreaticoduodenectomy (PD) and limited resection (segmental or wedge-shaped duodenectomy) ^(2)^, but the optimal surgical method remains controversial. An endoscopic examination reveals a submucosal tumor morphology, which makes the preoperative diagnosis difficult in many cases. A variety of surgical techniques are viable, depending on the size and location of the tumor.

## Case Report

The mean age was 63.7 years old, and the patients were five males and one female. The initial symptoms were bleeding from the tumor in five cases and hematemesis in one case.

**Figure 1.**
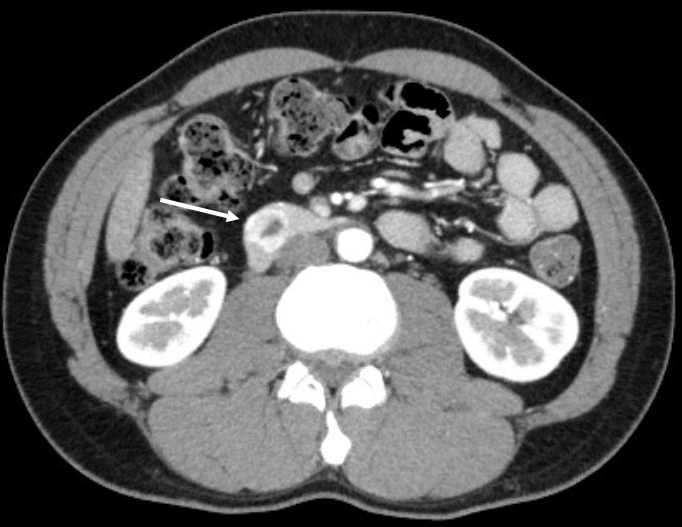
(Case 1) Contrast-enhanced computed tomography showed a heterogeneous tumor in the second portion of the duodenum (arrow).

**Figure 2.**
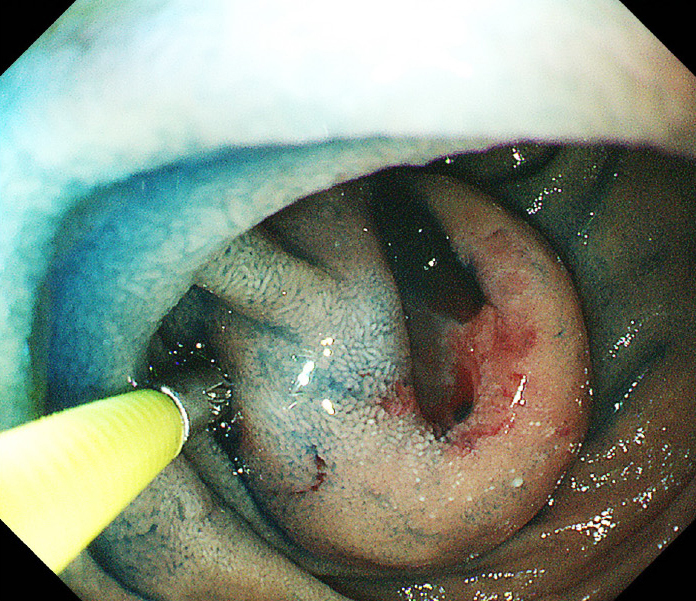
(Case 1) Esophagogastroduodenoscopy showed a tumor with ulceration in the second portion of the duodenum, which was definitely diagnosed by a biopsy.

Computed tomography (CT) showed an enhanced mass in the duodenum in all patients (Figure 1). Esophagogastroduodenoscopy (EGD) revealed a submucosal tumor without ulceration in one case and submucosal tumor with ulceration in five cases. With respect to the preoperative diagnosis, one patient was definitely diagnosed with dGIST by a biopsy (Figure 2), and five patients were diagnosed with suspected dGIST by EGD (Table 1).

**Table 1.** Clinical Characteristics and Tumor Location.

Case	Age (year)	Sex	Initial symptom	Findings of enhanced CT	Findings of EGD	Preoperative diagnosis	Location	Precise mural location of the duodenum
1	43	M	Bleeding	Enhanced mass	SMT with ulceration	GIST	Third	Anti-mesenteric
2	75	F	〃	〃	SMT	S/O GIST	〃	Anti-mesenteric
3	83	M	Hematemesis	〃	SMT with ulceration	〃	〃	Mesenteric
4	57	M	Bleeding	〃	〃	〃	〃	Mesenteric
5	70	M	〃	〃	〃	〃	Second	Anti-mesenteric
6	54	M	〃	〃	〃	〃	Fourth	Mesenteric

M: male, F: female, CT: computed tomography, SMT: submucosal tumor, EGD: esophagogastroduodenoscopy, S/O: suspect of, GIST: gastrointestinal stromal tumors

The tumor locations were the third portion in four cases, in second portion in one and fourth portion in one. With respect to the precise mural location of the duodenum, three cases were the mesenteric, and three cases were the anti-mesenteric part (Table 1).

**Figure 3.**
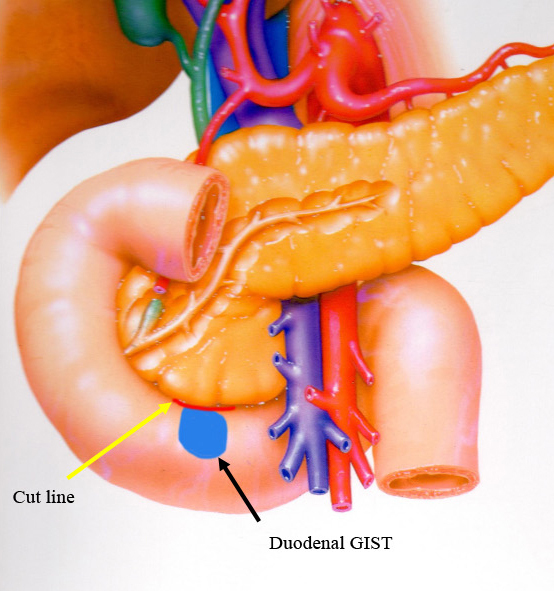
Wedge resection of duodenal gastrointestinal stromal tumors (dGISTs). dGIST of on the side of the pancreas (black arrow). Cut line between the duodenum and the pancreas (yellow arrow).

All patients underwent open surgical procedure without intraoperative endoscopy. They underwent duodenal wedge resection with primary sutures (Table 2). While three lesions were located on the side of the pancreas, wedge resection and direct suturing were possible (Figure 3).

**Table 2.** Surgical Procedure and Histopathological and Immunohistochemical Findings.

Case	Surgical operation	Approach	Intraoperative endoscopy	Size	Mitotic count	TNM	Stage	Resectional status	Tumor ulceration	KIT	CD34	S100	Desmin
1	Wedge resection with suturing	Open	None	30 mm	≧6/5 mm^2^	T2N0M0	IIIB	R0	(+)	(+)	(+)	(−)	(−)
2	〃	〃	〃	25 mm	≦5/5 mm^2^	T2N0M0	I	R0	(−)	(+)	(+)	(−)	(−)
3	〃	〃	〃	35 mm	≦5/5 mm^2^	T2N0M0	I	R0	(+)	(+)	(+)	(−)	(−)
4	〃	〃	〃	29 mm	≦5/5 mm^2^	T2N0M0	I	R0	(+)	(+)	(−)	(−)	(−)
5	〃	〃	〃	38 mm	≦5/5 mm^2^	T2N0M0	I	R0	(+)	(+)	(−)	(+)	(−)
6	〃	〃	〃	75 mm	≦5/5 mm^2^	T3N0M0	II	R0	(+)	(+)	(+)	(−)	(−)

R0: microscopically negative transection margins

The medium tumor size was 38.7 mm, with diameters ranging from 25 to 75 mm. Five patients were T2, and one was T3. The pathological stages were I in four patients, II in one, and IIIB in one. All patients underwent microscopically negative transection margins (R0) resection (Table 2).

All patients were discharged 12.8 days (10-15 days) postoperatively without complications, such as pancreatic fluid fistulae, suture failure or anastomotic stenosis. One case with stage IIIB disease received adjuvant chemotherapy with imatinib for 3 years. The median postoperative follow-up was 75 months (42-188 months), and all patients were alive without recurrence (Table 3).

**Table 3.** Adjuvant Chemotherapy and the Outcome and Survival.

Case	Postoperative complication	Postoperative stay (day)	Adjuvant chemotherapy	Recurrence	Status
1	None	11	Imatinib	None	3y6m alive
2	〃	13	None	〃	5y alive
3	〃	14	〃	〃	6y4m alive
4	〃	10	〃	〃	6y3m alive
5	〃	14	〃	〃	12y alive
6	〃	15	〃	〃	15y8m alive

## Discussion

The median age of the patients with dGIST is 56 years old, with a slight male preponderance of (54% vs. 46%) ^(1)^. Similar to a previous report ^(3)^, in the present study, dGISTs tended to cause intermittent bleeding, and the presence of duodenal or small bowel GISTs should be considered in patients without a source of bleeding in the stomach or colon. Since gastrointestinal bleeding was the most frequent initial symptom, EGD was routinely the method for the detection and verification of dGIST, which usually appears as a submucosal swelling with or without mucosal ulceration. Only one case was diagnosed by a biopsy, with five cases not diagnosed due to concerns about bleeding from the tumor. On contrast-enhanced CT, dGISTs appear as well-defined, heterogeneously enhanced, hypervascular masses with prominent feeding arteries and intra- or extramural growth ^(4)^. In our cases, contrast-enhanced CT showed an enhanced mass in all cases and proved very useful for preoperative planning of surgical treatment.

dGISTs have been observed to most frequently involve the second portion (51.6%), followed by the third (17.7%), fourth (16.1%), and first portions (14.6%) ^(5)^. GIST of the ampulla of Vater is extremely rare, and according to the review of Kobayashi et al., only 12 cases had been described as of 2014 ^(6)^.

Due to the anatomical specificity of dGISTs, various surgical techniques are employed, ranging from local resection to PD, depending on the localization, size, and nature of the tumor.

Wedge resection has been reported to be applicable to tumors in the vicinity of the ampulla of Vater when combined with intraoperative endoscopy or direct visual wedge resection through the duodenal mucosa and suture closure of the duodenum in the short axis for tumors of extramural growth or small intramural or intraductal growth of <2 cm ^(7)^.

A good knowledge of the anatomy, gentle handling of the tissue and careful dissection of the duodenal wall from the inferior border of the pancreas, meticulous hemostasis and understanding the possible options for duodenal reconstruction are mandatory for achieving a successful outcome. In our cases, we performed wedge resection with primary suturing in all cases. In three cases, dGISTs were located on the side of the pancreas, so we carefully dissected the duodenal wall from the inferior border of the pancreas and performed wedge resection of the dGIST with primary suturing, allowing us to perform minimally invasive, function-preserving surgery.

**Figure 4.**
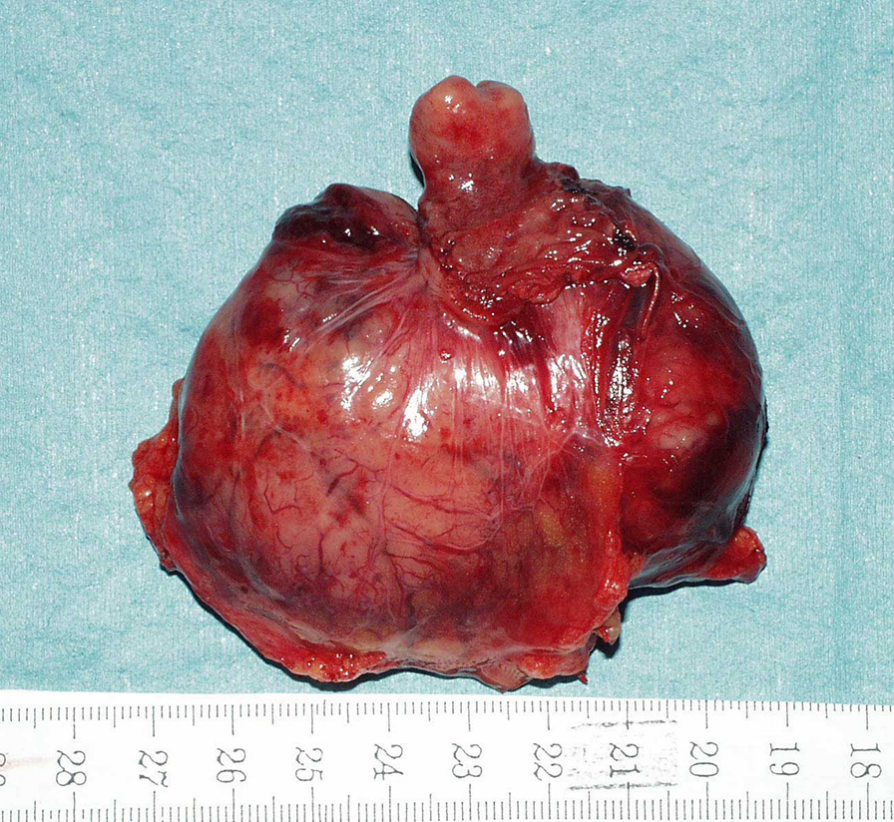
(Case 6) Resected specimen with wedge resection (R0).

After wedge resection, if the tumor base is large and there is concern about stenosis after simple closure, duodenal repair with a stemmed jejunal patch or double tract reconstruction with Roux-en-Y reconstruction may be necessary. In Case 6, the tumor was located on the side of the pancreas and was 75 mm in diameter, but it had an extramural growth type, and its base was small. Therefore, we performed wedge resection and sutured directly in the short axial direction without using a small patch, and no postoperative stenosis occurred (Figure 4). All of the present cases underwent R0 resection with an excellent disease-free survival and no local recurrence.

Several studies have shown that adjuvant imatinib treatment significantly improved the recurrence-free survival ^(8), (9)^. In our cases, Case 1 with stage IIIB disease received adjuvant imatinib treatment for 3 years and experienced no disease recurrence.

## Article Information

### Conflicts of Interest

None

### Author Contributions

TU analyzed the data and wrote the manuscript. TU, IE, and MK performed the surgery, and HA and MK helped draft the manuscript. TN, NW, OY, HA, and MK participated in revising the manuscript critically. All authors declare that they contributed to this article and that they read and approved the final version.

### Approval by Institutional Review Board (IRB)

21-CR01-159

Ethics Committee, Mitoyo General Hospital

### Availability of Data and Materials

All data generated or analyzed during this study are included in this published article.

### Consent for Publication

Consent to publish was obtained from the patients.

## References

[1] Winfield RD, Hochwld SN, Vogel SB, et al. Presentation and management of gastrointestinal stromal tumors of the duodenum. Am Surg. 2006;72(8):719-22; discussion 722-3. 16913316

[2] Bourgouin S, Hornez E, Guiramand J, et al. Duodenal gastrointestinal stromal tumors (GISTs): argument for conservative surgery. J Gastrointest Surg. 2013;17(3):482-7. 23229887 10.1007/s11605-012-2075-3

[3] Johnston FM, Kneuertz PJ, Cameron JL, et al. Presentation and management of gastrointestinal stromal tumors of the duodenum: a multi-institutional analysis. Ann Surg Oncol. 2012;19(11):3351-60. 22878613 10.1245/s10434-012-2551-8

[4] Popivanov G, Tabakov M, Mantese G, et al. Surgical treatment of gastrointestinal stromal tumors of the duodenum: a literature review. Transl Gastroenterol Hepatol. 2018;3:71. doi: 10.21037/tgh.2018.09.04. eCollection 2018. 30363779 PMC6182027

[5] Gu L, Khadaroo PA, Chen M, et al. Surgical management and outcomes of duodenal gastrointestinal stromal tumors. Acta Gastroenterol Belg. 2019;82(1):11-8. 30888748

[6] Kobayashi M, Hirata N, Nakaji S, et al. Gastrointestinal stromal tumor of the ampulla of Vater: a case report. World J Gastroenterol. 2014;20(16):4817-21. 24782637 10.3748/wjg.v20.i16.4817 PMC4000521

[7] Zhou Y, Wang X, Si X, et al. Surgery for duodenal gastrointestinal stromal tumor: a systemic review and meta-analysis of pancreaticoduodenectomy versus local resection. Asian J Surg. 2020;43(1):1-8. 30853211 10.1016/j.asjsur.2019.02.006

[8] Joensuu H, Wardelmann E, Sihto H, et al. Effect of KIT and PDGFRA mutations on survival in patients with gastrointestinal stromal tumors treated with adjuvant imatinib: an exploratory analysis of a randomized clinical trial. JAMA Oncol. 2017;3(5):602-9. 28334365 10.1001/jamaoncol.2016.5751 PMC5470395

[9] Hohenberger P, Eisenberg B. Role of surgery combined with kinase inhibition in the management of gastrointestinal stromal tumor (GIST). Ann Surg Oncol. 2010;17(10):2585-600. 20407930 10.1245/s10434-010-1053-9

